# The efficacy of vector-proof accommodation for the protection of livestock against *Culicoides* biting midges

**DOI:** 10.1186/s13071-025-06736-9

**Published:** 2025-03-14

**Authors:** Simon King, Melanie Nicholls, Jake Scales, Simon Gubbins, Paul Pearce-Kelly, Stefan Saverimuttu, Sarah Forsyth, Marion England

**Affiliations:** 1 The Pirbright Institute, Ash Road, Woking, Surrey GU24 0NF UK; 2 Zoological Society of London, Regents Park, London, NW1 4RY UK

**Keywords:** *Culicoides*, Bluetongue, Vector-proof accommodation

## Abstract

**Background:**

Bluetongue virus serotype 3 emerged in northern Europe and the UK for the first time in 2023, causing significant losses of domestic ruminants. Vector-proof accommodation is considered a potential control measure to reduce host-vector contact, but this has not been investigated in northern Europe. This study investigates the efficacy of vector-proof accommodation to protect livestock from *Culicoides* biting midges in the UK.

**Methods:**

Four identical stables were fitted with various levels of vector-proofing, using readily available materials, and a CDC light trap in each. Two further CDC light traps were set outside the stables. For 19 nights during June and July 2024, two pygmy goats were placed into each stable and traps were run until the following morning to collect *Culicoides*. Trap catch comparisons for total *Culicoides*, *Avaritia* females and *Culicoides obsoletus/scoticus* were analysed using negative binomial generalised linear models.

**Results:**

The use of brushes around closed doors resulted in a 14-fold reduction in the number of *Culicoides* collected from indoor traps. There was an additional effect of installing fine mesh over slatted windows, but this was not significant. Housing animals without shutting the door had no effect on the number of *Culicoides* collected compared to outside. Blood meal analysis confirmed *Culicoides obsoletus/scoticus* collected from inside stables were feeding on the goats.

**Conclusions:**

This study has shown that small animal housing can be cheaply and easily fitted with readily available vector-proof materials to effectively protect a small number of animals from *Culicoides*. The efficacy and suitability of vector-proof accommodation may vary with time of year, and consideration needs to be given to the welfare implications of housing animals during the summer.

**Graphical Abstract:**

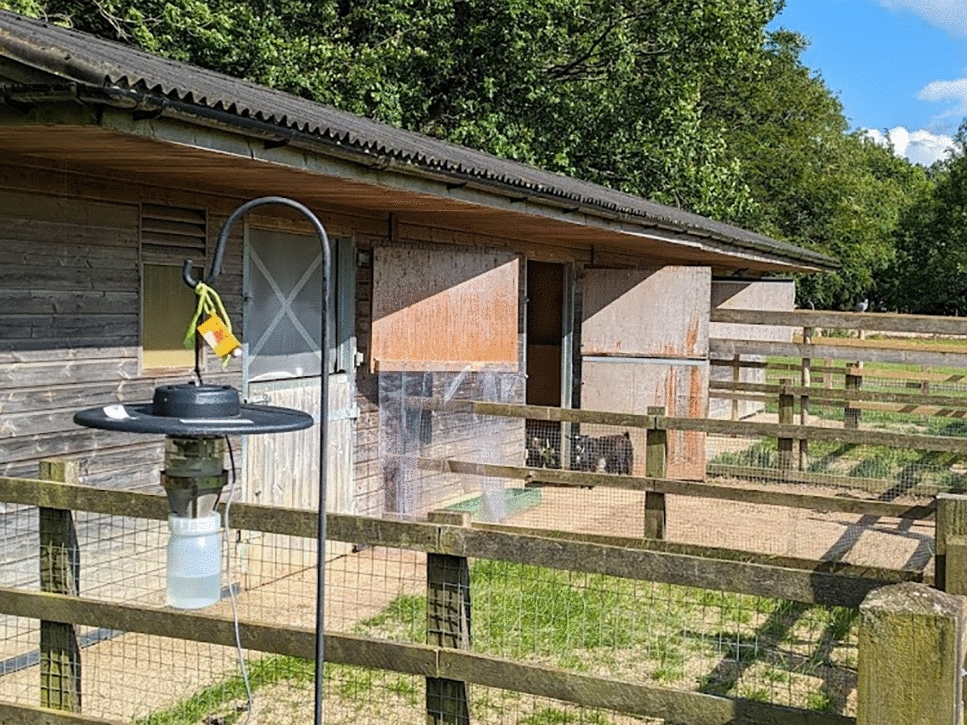

**Supplementary Information:**

The online version contains supplementary material available at 10.1186/s13071-025-06736-9.

## Background

Bluetongue virus (BTV) is an *Orbivirus* in the family *Sedoreoviridae* and causes bluetongue disease in wild and domestic ruminants, including cattle, sheep, goats and camelids. BTV is spread through the bites of infected *Culicoides* midges (Diptera: Ceratopogonidae). Bluetongue was historically a disease of the tropics, but since 1998, there have been multiple incursions of several different serotypes into Europe [1]. Most notably, in 2006, bluetongue virus serotype 8 (BTV-8) emerged in northern Europe for the first time causing substantial economic losses to the farming industry [2]. The scale of this outbreak was unprecedented and represented a significant geographical expansion of bluetongue disease. The outbreak of BTV-8 was largely brought under control through vaccination, with the UK reclaiming freedom from disease in 2011 [3].

In September 2023, bluetongue virus serotype 3 (BTV-3) emerged for the first time in northern Europe in The Netherlands and spread very rapidly across large parts of the country and into Belgium and Germany [4]. By November 2023, BTV-3 had also spread to the UK, probably via the windborne incursion of infected *Culicoides* biting midges [5]. The virus successfully overwintered in northern Europe and re-emerged in Germany, Belgium and The Netherlands in 2024. At the end of August, it re-emerged in the UK and throughout 2024 spread to many other European countries, including Portugal and Italy in the south and Denmark and Norway in the north [6].

With the sudden emergence of BTV-3 in northern Europe in autumn 2023, protection of livestock from exposure to infected midges has become a critical issue for both livestock keepers and policy makers attempting to reduce occurrence of the virus. Vaccines against BTV-3 have been developed which claim to reduce the clinical severity of BTV-3 infection and reduce mortality [7]. The efficacy of these vaccines in the field is unknown, and they are currently being used under emergency licence in some affected countries including the UK. Whilst vaccines have the potential to provide protection against severe clinical disease, other methods to reduce vector-host contact may also need to be considered. Direct control of *Culicoides* is currently challenging because of their huge local abundance on farms and their ubiquitous use of a wide range of breeding habitats [8]. There is no evidence that using insecticides and/or repellents on farms and directly on animals reduces BTV transmission in the field [8]. Vector-proof accommodation requires that all *Culicoides* are excluded from animal housing using physical barriers such as fine mesh and closed sealed doors/windows. The use of vector-proof accommodation to protect animals has been previously explored, notably for the protection of horses against African horse sickness virus (a related *Culicoides*-borne virus) in endemic regions of South Africa, which observed that housing of horses protected them from exophagic vector species but increased attack by endophagic species [9]. This strategy was employed during the 2020 outbreak of African horse sickness in Thailand but also highlighted the animal welfare implications of such housing for long periods of time in hot and humid conditions [10].

Vector-proof accommodation does not seek to reduce overall transmission of the virus at a regional scale; rather it may provide a means to protect individual animals at a local scale [11]. This study investigates the efficacy of different levels of vector-proof accommodation to protect livestock against *Culicoides* biting midges. Readily available vector-proofing materials were fitted to standard animal stables such that these could be easily replicable by livestock keepers on their own animal housing.

## Methods

### Field setup

**Fig. 1:**
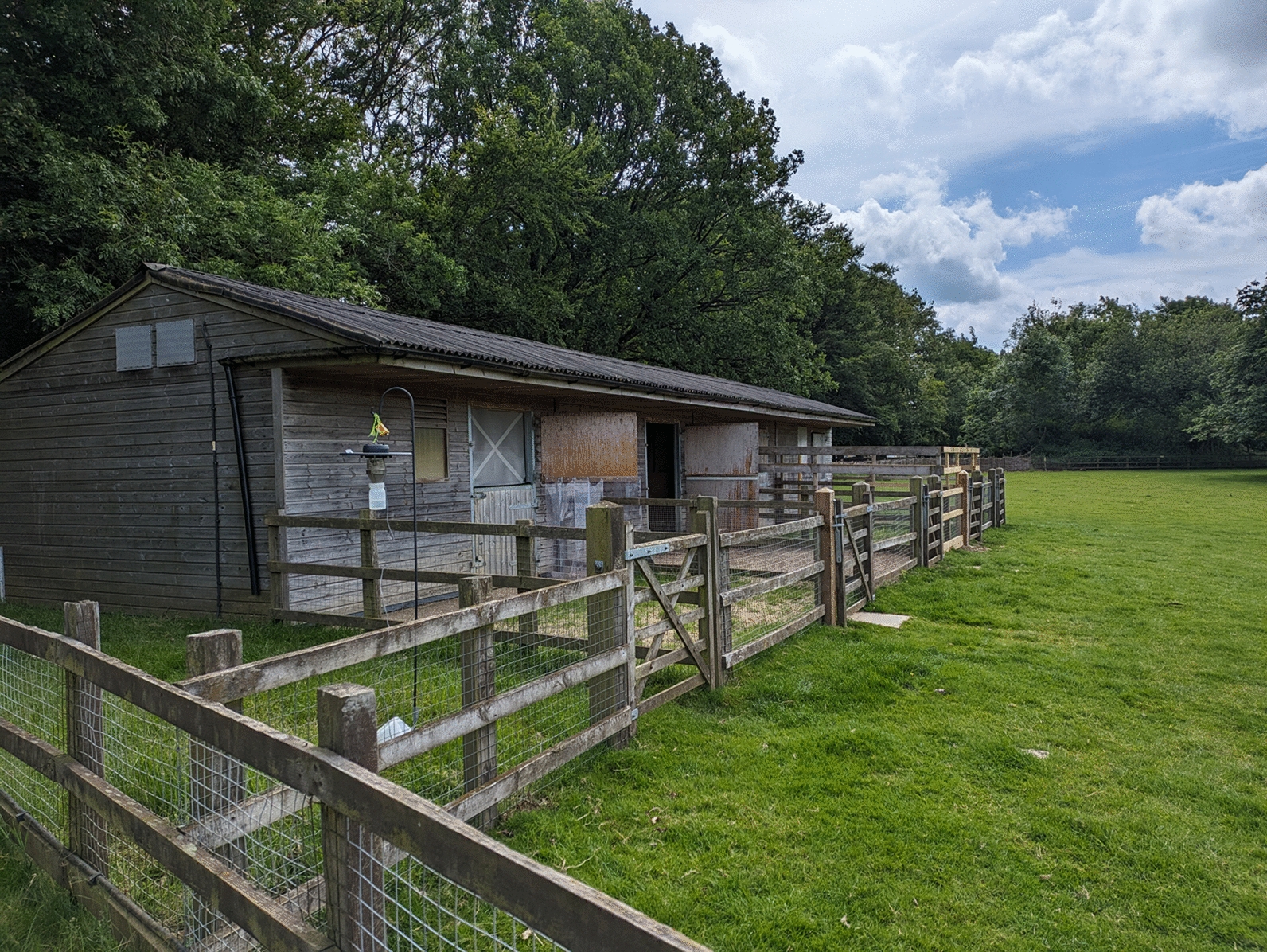
Stable block used to investigate vector-proof accommodation at Whipsnade Zoo in the UK

**Fig. 2:**
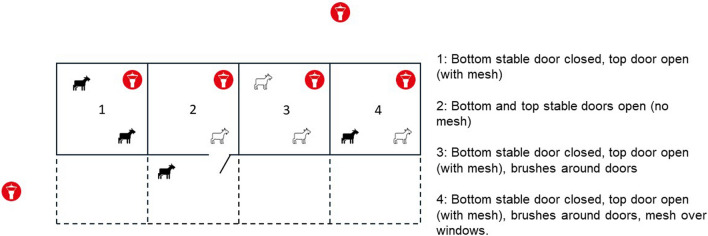
Setup of vector-proof accommodation across four stables (numbered 1–4) at Whipsnade Zoo. Each stable was randomly allocated two pygmy goats on each trapping night. The red symbols indicate the placement of CDC light traps inside and outside the stables. The solid lines indicate the stable walls and dotted lines indicate the paddock area of each stable. Only goats in stable 2 had access to the paddock area

To test the efficacy of vector-proof accommodation against *Culicoides* biting midges, a single stable block consisting of four identical stables at Whipsnade Zoo, a ZSL Conservation Zoo, was used. Whipsnade Zoo is in a rural location in Bedfordshire (51°50′51.72"N, 0°32′22.29"W), and the stable block used was situated close to the petting farm exhibit (Fig. 1). Each stable was allocated one of four levels of vector-proofing, ranging from none to full vector-proofing. Vector-proofing was achieved through a combination of draft excluder brushes (e.g. Trintion 100 cm Door Brush Aluminium Draught Excluder Strip, Amazon.co.uk, Fixman 447234 Garage Door Brush Strip 50 mm Bristles, Amazon.co.uk, TA-VIGOR Brush Weather Stripping Adhesive Felt Door Seal, Amazon.co.uk), expanding foam (Rapide Bond & Fill Expanding Foam Filler, Amazon.co.uk), fine stainless steel mesh with hole aperture 0.5 mm (Midge Mesh, Robinson Wire Cloth Ltd) and duct tape (Duck Tape Original, Amazon.co.uk) (Fig. S1). Stables were numbered 1 to 4, and stable 2 was selected to have no vector-proofing with the door remaining open at all times as swallows (*Hirundo rustica*) were nesting in this stable. Stable 1 had no vector-proofing, but the door was kept closed. Stable 3 had the door closed, brushes fitted around the doors to cover gaps up to 5 cm in size and expanding foam to fill any gaps in the walls and ceiling. Stable 4 had the door closed, brushes fitted around the doors to cover gaps up to 5 cm in size, expanding foam and duct tape to cover any gaps in the walls and ceiling, and stainless steel midge mesh fitted over slatted windows (Fig. 2). All stables had a small open window covered with wooden slats but these were left uncovered in stables 1, 2 and 3. For all stables, to enable airflow for animal welfare, the top stable doors were left open and replaced by stainless steel midge mesh (with the exception of stable 2).

A single UV miniature CDC light trap (John Hock, USA) was hung in the back-right corner of each of the four stables. Additionally, a single trap was placed outside both the front and back of the stables. A temperature datalogger was fitted alongside each trap (TinyTag, Gemini, UK).

Between 4 June 2024 and 2 July 2024, two West African pygmy goats (*Capra hircus*) were randomly allocated to each of the four stables (total of eight pygmy goats) and placed in the stables from 4 p.m. to 8 a.m. for a total of 19 nights. Pygmy goats were selected as the host species as they were already present on site and could be easily moved into and out of the stables daily. They were kept in pairs for welfare reasons and were randomly allocated to each stable on each trapping day to control for individual variation in attractiveness to *Culicoides*. When the goats entered the stables, all six CDC light traps were turned on. When the goats left the stables the following morning, the traps were switched off and emptied. Collected insects were transferred immediately into 70% ethanol for later identification. The stables were cleaned and then left empty during the day with doors closed, apart from stable 2 where the paddock door was left permanently open. Goats were only housed in the stables, and therefore trapping was only carried out, on nights when the temperature in the stables was < 23 °C at 4 p.m. for animal welfare reasons.

### *Culicoides* processing

All collected *Culicoides* from the 19 trapping nights were identified morphologically to species level under a light dissecting microscope using existing keys [12, 13]. Female specimens of *Culicoides obsoletus* Meigen 1818 and *Culicoides scoticus* Downes and Kettle 1952 cannot be distinguished morphologically and so were recorded as '*Culicoides obsoletus/scoticus*'. Females were further categorised according to the morphological presentation of their abdomens as unpigmented, pigmented, blood-fed or gravid [14]. Blood-fed females were separated and further processed to identify the host species on which they had fed. Non-*Culicoides* were stored in 70% ethanol as by-catch but were excluded from further analysis in this study.

### Blood meal analysis

Blood meal analysis was conducted according to a previously described protocol [15]. Briefly, abdomens of blood-fed individuals were homogenised and extracted using the Qiagen DNeasy Blood and Tissue kit (Qiagen), according to manufacturer’s instructions. PCR was performed using Qiagen HotStarTaq (Qiagen) in a final volume of 25 µl. Purified PCR products were sequenced bi-directionally using M13 primers and sequences assembled in SeqMan Pro v 17.3.0 (DNASTAR). DNA sequences were assigned to a host species if they displayed a match of > 98% compared to all available GenBank sequences using BLAST.

### Data analysis

Data were analysed using negative binomial generalised linear models (GLMs) implemented with the MASS and multicomp packages in R version 4.4.0 [16]. Three models were constructed with total *Culicoides*, subgenus *Avaritia* females and *C. obsoletus/scoticus* females as dependent variables, respectively, and trap location and temperature as independent variables. Temperature was included in the models as daily mean temperature (as measured by the temperature datalogger for the trap) on the day that the trap was set.

## Results

### *Culicoides* collection

**Fig. 3:**
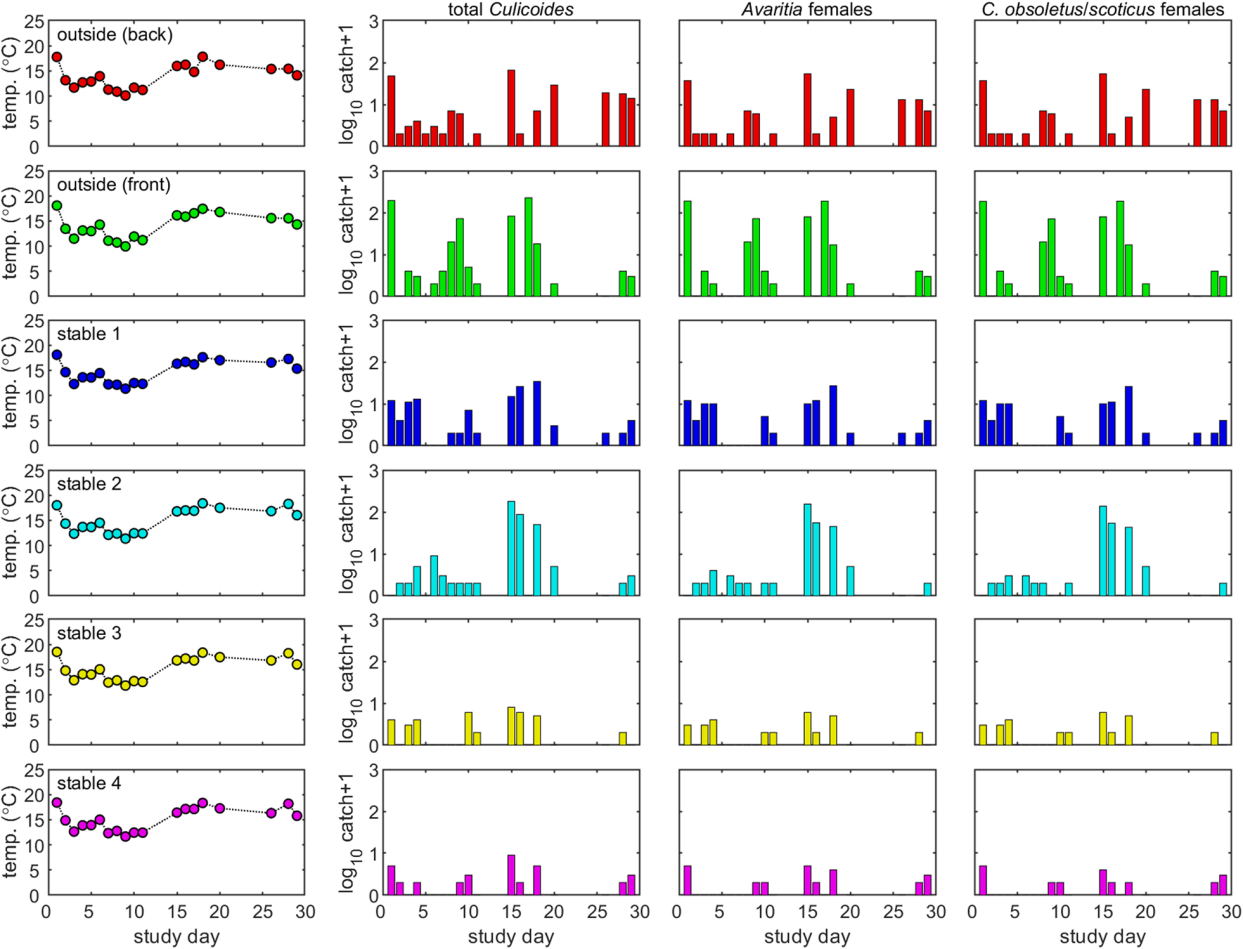
Total (log10) catches of *Culicoides*, *Avaritia* females and *C. obsoletus/scoticus* females across study days for each trap (outside back = red, outside front = green, stable 1 = dark blue, stable 2 = light blue, stable 3 = yellow and stable 4 = pink). Column 1 shows how the temperature measured at each trap varied across the study period

A total of 1362 *Culicoides* of 14 different species were collected during the study period, both inside and outside the stables. The greatest diversity of species was seen from the outside traps and from the open stable (stable 2), with six species being found within the closed stables (stables 1, 3 and 4). The results are shown in Fig. 3 and the total number and species of *Culicoides* collected from each trap are shown in Table 1. The total number of males and female parity status is given in Table 2; 58.0% were unpigmented, 31.3% were pigmented, 3.5% were blood-fed and 1.5% were gravid females; 5.7% were male. One *C. obsoletus/scoticus* female was damaged and could not be assigned a parity status but was included in the analyses, and one male *Culicoides* was damaged and could not be identified to species level but was included in the analyses for total *Culicoides.*

**Table 1:** Total number of *Culicoides* collected from inside and outside traps at Whipsnade Zoo. Vector species of BTV indicated by ǂ

Subgenus	Species	Trap					
		Stable 1	Stable 2	Stable 3	Stable 4	Outside (front)	Outside (back)
*Avaritia*	*C. obsoletus/scoticus ǂ*	115	265	30	20	567	167
	*C. chiopterus ǂ*	2	22		3	6	1
	*C. dewulfi ǂ*					1	
*Culicoides*	*C. pulicaris ǂ*	5	13			19	12
	*C. punctatus ǂ*		14			29	12
*Oecacta*	*C. brunnicans*	1	15		1	1	18
	*C. vexans*		1				
*Beltranmyia*	*C. circumscriptus*						1
*Monoculicoides*	*C. parroti*		1				
*Sensiculicoides*	*C. kibunensis*						4
	*C. poperinghensis*		9				
*Sylvaticulicoides*	*C. achrayi*	1		1			1
	*C. fascipennis*				1	1	2
Total		124	340	31	25	624	218

**Table 2:** Sex and parity status of *Culicoides* collected from each trap at Whipsnade Zoo

Sex	Parity	Trap					
		Stable 1 (%)	Stable 2 (%)	Stable 3 (%)	Stable 4 (%)	Outside front (%)	Outside back (%)
Female	Unpigmented	70 (56.5)	209 (61.5)	19 (61.3)	13 (52.0)	375 (60.0)	105 (48.4)
	Pigmented	16 (12.9)	85 (25.0)	0 (0)	5 (20.0)	230 (36.8)	89 (41.0)
	Gravid	0 (0)	1 (0.3)	0 (0)	0 (0)	9 (1.4)	10 (4.6)
	Blood-fed	10 (8.1)	25 (7.4)	2 (6.5)	1 (4.0)	6 (1.0)	4 (1.8)
Male		28 (22.6)	20 (5.9)	10 (32.3)	6 (24.0)	5 (0.8)	9 (4.1)
Total		124	340	31	25	625	217

As temperature increased, the number of *Culicoides* caught increased, with a 1 °C increase in temperature resulting in a 35% (95% confidence interval: 19–52%) increase in the number caught (Table S1). The temperatures within some stables were higher than others because of the orientation of the stable block regarding the sun, but temperature differences between trap locations were controlled for in the model.

**Fig. 4:**
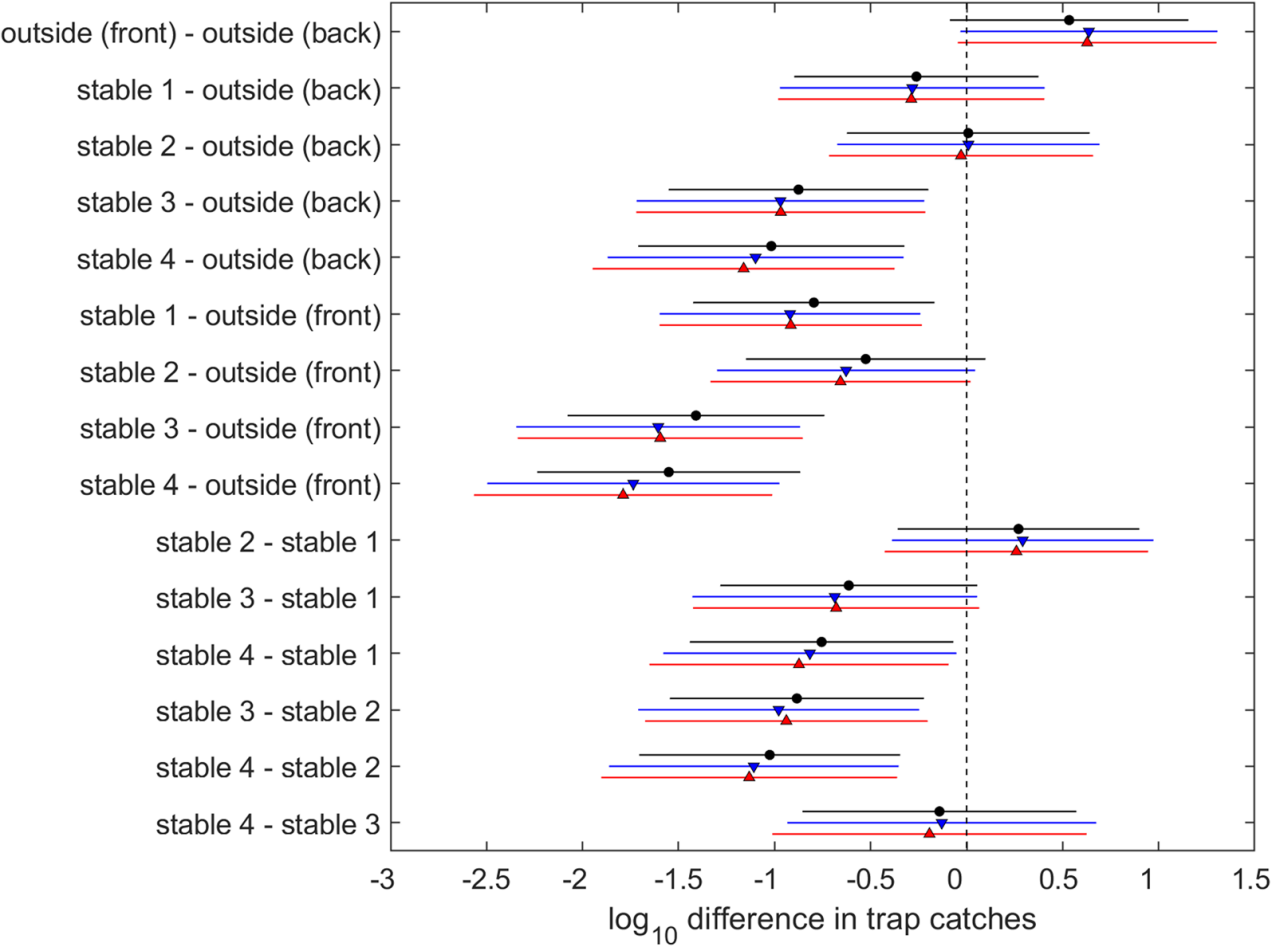
Difference in *Culicoides* collections (log_10_) between traps for total *Culicoides* (black circles), *Avaritia* females (red triangles) and *Culicoides obsoletus/scoticus* females (blue triangles)

There was no significant difference (*p* > 0.05) in the number of *Culicoides* collected from the two outside traps or between the open stable (stable 2) and either of the outside traps (Table S1). Therefore, housing the animals without shutting the stable door did not significantly reduce the numbers of *Culicoides* that the animals were exposed to. Additionally, no significant difference was observed between stable 1 and stable 2, suggesting that shutting the door did not lead to a significant reduction in *Culicoides* exposure compared to not shutting the door. However, fewer *Culicoides* were caught in stable 1 compared with the outside front trap (*p* < 0.01) but not the outside back trap (*p* = 0.13). Significantly fewer *Culicoides* were collected in both stables 3 (brushes only) and 4 (brushes and mesh) compared to both outside traps and stable 2 (open stable) (*p* < 0.01). Fewer *Culicoides* were also collected in stables 3 and 4 than stable 1 (door shut) but this was only statistically significant for stable 4 (*p* = 0.09 and *p* = 0.02, respectively). There were no statistically significant differences between the number of *Culicoides* collected from stable 3 and stable 4 (*p* = 0.99). On average, the outside traps collected 22.1 *Culicoides* per night. Vector-proofing of stables 3 and 4 resulted in a 14- and 17-fold reduction in the average number of *Culicoides* collected per night, respectively. These results are shown in Fig. 4.

Similar results for the effect of temperature and differences among trap location were obtained when considering only subgenus *Avaritia* females and *C. obsoletus*/*scoticus* females. This is because they comprised the vast majority of the *Culicoides* caught (Tables 1 and 2).

### Blood meal analysis

A total of 48 blood-fed (full and partial) *Culicoides* were collected during the study; 46 *C. obsoletus/scoticus*, 1 *Culicoides pulicaris* Linnaeus 1758 and 1 *Culicoides punctatus* Meigen 1804. Of these, sequences were successfully obtained from 13 specimens (27.1%) and matched to sequences in GenBank. Host bloodmeal was identified as *C. hircus* (goat) for 12 *C. obsoletus/scoticus* specimens, and one *C. punctatus* specimen, collected in an outside trap, had fed on *Ovis aries* (sheep), which were present in the adjacent paddocks. Blood-fed specimens were collected from all traps but there were notably more from the inside traps compared to outside: stable 1 (*n* = 7), stable 2 (*n* = 17), stable 3 (*n* = 1), stable 4 (*n* = 1), outside back (*n* = 3) and outside front (*n* = 4).

## Discussion

This study explored different levels of closed housing and vector-proofing as a potential means to protect livestock from *Culicoides* and the risk of bluetongue disease. It demonstrated that the use of effective sealing materials, such as brushes around doors, can significantly reduce the presence of *Culicoides* in stables, while the addition of fine mesh did not provide statistically significant additional benefits. Pygmy goats were used to bait the four stables, with the outside traps situated within close proximity to a range of domestic and wild animals, including various goats, sheep, camelids and birds. The trap located behind the stable block [outside (back)] was also adjacent to an enclosure housing Darwin’s rhea (*Rhea pennata*), a species which has been previously confirmed as a host for *Culicoides* at the zoo [15]. All six putative vectors of BTV-3 were collected in this study: *Culicoides obsoletus, C. scoticus, C. dewulfi* Goetghebuer 1936*, C. chiopterus* Meigen 1830*, C. pulicaris* and *C. punctatus* [17, 18]. The six vector species have been shown to be generalist feeders on mammalian species [15, 19] and feeding on goats was confirmed in 12 specimens of *C. obsoletus/scoticus.* The results indicate that housing animals in an open stable did not afford any protection against *Culicoides* biting, with *Culicoides* readily entering stables to bite. Indeed, more blood-fed *Culicoides* were found inside the stables compared to outside. Closing the stable doors reduced the number of *Culicoides* collected from inside stable 1 compared to the outside (front) trap but not when compared to the outside (back) trap or the open stable (stable 2). This was, therefore, not considered a sufficient measure to protect the animals inside the stable from being bitten.

Stables 3 and 4 were fitted with vector-proofing materials, and this significantly reduced the number of *Culicoides* collected, down to a maximum of six individual vectors collected in a single night. Stable 3 was vector-proofed such that gaps, up to around 5 cm in size, around doors were closed up using garage door brushes and draft excluder brushes cut to size. Large gaps in the eaves were blocked up with expanding foam. Stable 4 was secured in the same way as stable 3, but with the addition of midge mesh over the slatted window and duct tape over small holes in the walls. The vector-proofing materials used on stable 3 provided a cheap, readily available cost-effective solution and were shown to significantly reduce the number of *Culicoides* entering the stable to almost zero. The addition of stainless steel midge mesh on the window of stable 4 did not significantly enhance vector-proofing. Over the course of the study a total of 30 *Culicoides* were collected in stable 3 compared to 25 in stable 4. The stainless steel midge mesh was considerably more expensive than the other materials and was difficult to source because of the requirement of a 0.5-mm hole aperture. Whilst other materials such as a tight-weave fabric would offer a smaller hole aperture and be easier to obtain, they would have restricted airflow to a greater extent as well as having a lower durability. This would present a problem on a working farm environment with damage to the fabric negating the vector-proofing and causing financial and time costs in repairs or replacements.

The stables at either end of the block had additional slatted windows on the side walls, and to standardise the four stables, these were covered with stainless steel midge mesh. It was not necessary to use mesh for this purpose. Blocking off with any suitable material would have been sufficient for the purposes of the study. However, the use of midge mesh in place of closing the top stable door (Fig. S1) enabled greater airflow within the stables and was considered necessary for the welfare of the animals inside. In consultation with onsite vets and animal keepers, the decision was taken not to conduct the study on days when the temperatures in the stables exceeded 23 °C at 4 p.m., when the goats would have been moved inside the stables.

Animal welfare considerations played a large role in this study and are an important factor in the use of vector-proof accommodation. The decision was taken to leave all top stable doors open to enable greater air circulation, and for stables 1, 3 and 4, these areas were covered with midge mesh instead of closing the door. For larger animal housing, with inherently greater air movement, this may not be necessary. In this study, small fans were fitted to each stable which could be used to keep the animals cool. These were fitted so that they pointed away from the CDC light traps and angled down towards the animals. However, in the end it was not necessary to turn these on at any stage during the study. *Culicoides* are unable to actively fly at wind speeds > 3 m/s [20]. Therefore, we believe the addition of fans is beneficial both from a welfare point of view and for protection against vector biting. The use of fans would also allow all doors to be closed whilst minimising impact on animal welfare in terms of ventilation and air circulation. However, this was not a method tested in our study, partly because of the unknown impact of greater air circulation on our ability to collect *Culicoides* using CDC light traps. The study was conducted prior to the re-emergence of BTV-3 in the UK, and therefore on hotter evenings, animal welfare considerations always outweighed disease risk and animals were not placed in the stables. If BTV-3 was circulating in the area at the time of the study, the use of the fans to cool the animals in the stable may have enabled them to be placed within the vector-proof accommodation. This decision would ultimately be down to livestock keepers, weighing up the disease risk against animal welfare considerations. Notably, in The Netherlands, it has been shown that the addition of fans and increased natural ventilation has been beneficial in reducing the occurrence of BTV [4, 21], although ceiling fans were not shown to afford any additional protection against *Culicoides* for horses in South Africa [9]. More extensive ventilation systems may enable animals to be housed in closed facilities during periods of warmer weather as the internal temperature of the facility could be more easily controlled. Such systems would inevitably incur a large installation and running cost though, which may only be feasible for holdings with high-value animals such as artificial insemination/breeding centres.

Alongside the investigation into vector-proofing, this study also provides valuable data on the propensity of BTV vectors to enter animal housing. This has previously been investigated in northern Europe, where both exophilic and endophilic biting behaviours have been observed, with no clear species preferences [22–26]. Whilst one study in The Netherlands found that *C. pulicaris* does not readily enter livestock accommodation [27], another study conducted in Germany has found evidence to the contrary [28]. In this study, all vector species were collected from both inside and outside stables, except for *C. dewulfi*, which was not collected from inside the stables. However, only a single *C. dewulfi* was collected during the entire study so no conclusion can be drawn about their preference for outdoor vs. indoor biting. *Culicoides punctatus* was not found in any of the closed stables but *C. obsoletus/scoticus* was found in all stables and was the most abundant species collected. Their presence in all stables may reflect their greater propensity to enter animal housing compared to other species or be a reflection of their greater presence overall and their attraction towards the CDC light traps. However, it appears that even during the summer months, they will move inside to find a blood meal, despite considerable physical barriers. The closed stables were kept closed apart from when animals were being moved in and out, resulting in the possibility that *Culicoides* entered the stables during this time, although animal movement occurred at times of the day when *Culicoides* are not usually active.

The stabling of animals and vector-proof accommodation have been investigated previously both in the context of protecting horses against African horse sickness virus (AHSV) and to protect cattle and sheep against BTV. In South Africa, vector-proof stabling using gauze provided effective protection by reducing the presence of *Culicoides* inside stables 14-fold, the same as found in this study when brushes were used [9]. However, in South Africa both endophilic (*Culicoides bolitinos* Meiswinkel 1989) and exophilic (*Culicoides imicola* Kieffer 1913) species are involved in transmission of AHSV, meaning that the gauze is essential to exclude those with a propensity to move indoors to feed. Screening horse stables in the UK with untreated mesh (1.6-mm aperture) also significantly decreased the number of *Culicoides* trapped by nearly fivefold compared to an unscreened stable [29]. The protective effect of holding cattle in pens with roofs and/or walls was mixed in a study conducted in Australia [30]. Numbers of the endophagic *Culicoides actoni* Smith 1929 increased in more enclosed pens, whereas there was a reduction in the numbers of other species. Similar species differences were observed in another Australian study looking at the effect of pen covers on *Culicoides* abundance [31]. A further study conducted in Australia investigated the use of physical barriers (tarpaulins with and without insecticide treatment) around pens containing sheep [32]. Whilst some protective effect was found with insecticide-treated barriers, the structures used cannot be classed as ‘housing’ as they were not roofed. In Canada, a study showed that unscreened stables would not protect horses against *Culicoides* bites, although only 6% of *Culicoides* collected were from traps located inside [33]. There are likely multiple factors that influence movement into livestock accommodation, not least the time of year and meteorological factors. Further work is required to determine whether species-specific behaviours vary across space and time and whether this will impact the level of vector-proofing required.

In this study, CDC light traps were used as a measure of *Culicoides* presence within and outside of housing. There are a number of limitations to this approach, for example not all *Culicoides* species are equally attracted to the light bait. It is considered that *C. chiopterus* are underrepresented in light traps, and as such their role as BTV vectors may be underestimated [34]. Indeed, the presence of the hosts in the stables may have diverted midges away from the trap, such that the goats were experiencing greater biting pressure than the light traps indicated. *Culicoides* are thought to seek a resting habitat following feeding, and whilst blood-fed specimens were collected within the traps, there were potentially more *Culicoides* present in the stables, which were no longer exhibiting host-seeking behaviour after feeding on the goats and therefore not collected in the traps. It is also possible that some of the goats were more attractive to *Culicoides* than others because of individual characteristics or behaviour. However, we controlled for this in the study by randomly allocating the goats to each stable each night, thereby reducing any bias. Given the standardisation of hosts and traps across the stables, and the greater numbers of *Culicoides* collected in the outside traps, an inference of relative protection remains valid.

## Conclusions

This study has shown the efficacy of different levels of vector-proofing against *Culicoides* biting midges. It has shown that readily available, cheap materials can be used to significantly reduce entry of *Culicoides*, and specialised midge mesh does not afford greater protection. However, this study vector-proofed small stables, whereas typical animal housing for cattle in the UK involves large barns, sometimes with open sides. For some large barns, it may be possible to fit materials such as those used in this study to close large gaps. The use of midge mesh with aperture ≤ 0.5 mm will effectively exclude *Culicoides* and may be appropriate where the costs and labour of fitting outweigh the risks from BTV. Additionally, in some instances, it may be a more practical measure than using brushes. This study has shown that if smaller housing is available, it can be cheaply and easily fitted with vector-proof materials to effectively protect a small number of animals. This would be an effective measure to protect against BTV for smallholdings or to protect individual animals on a larger farm, such as breeding stock. The temperament of the animal and, particularly for herd/flock animals, the welfare implications of holding them individually or in small groups along with weather conditions need to be considered against the requirement for protection against BTV. The time of year may also have an impact on the decision to move animals into vector-proof accommodation and this requires further study.

## Supplementary Information

Supplementary Material 1. Figure S1. Materials used to vector-proof the stables and CDC light trap within a stable. A: Five-centimetre garage door brushes, B: fine stainless steel mesh, C: CDC light trap within stable, D: felt door seal andduct tape, E: expanding foam and fine stainless steel mesh

Supplementary Material 2. Table S1. Estimated coefficients in the negative binomial GLMs for numbers of *Culicoides* caught each trap night.

## Data Availability

Raw data provided within the manuscript are available upon request from the corresponding author.

## Abbreviations

CDC: Centers for Disease Control and Prevention
BTV: Bluetongue virus
ZSL: Zoological Society of London
UV: Ultraviolet
GLM: Generalised linear model
AHSV: African horse sickness virus
