# Supplementary figures and images for: The efficacy of vector-proof accommodation for the protection of livestock against Culicoides biting midges
Source: Parasit Vectors. 2025 Mar 14;18:108. doi: 10.1186/s13071-025-06736-9 (PMC11909808; doi:10.1186/s13071-025-06736-9)

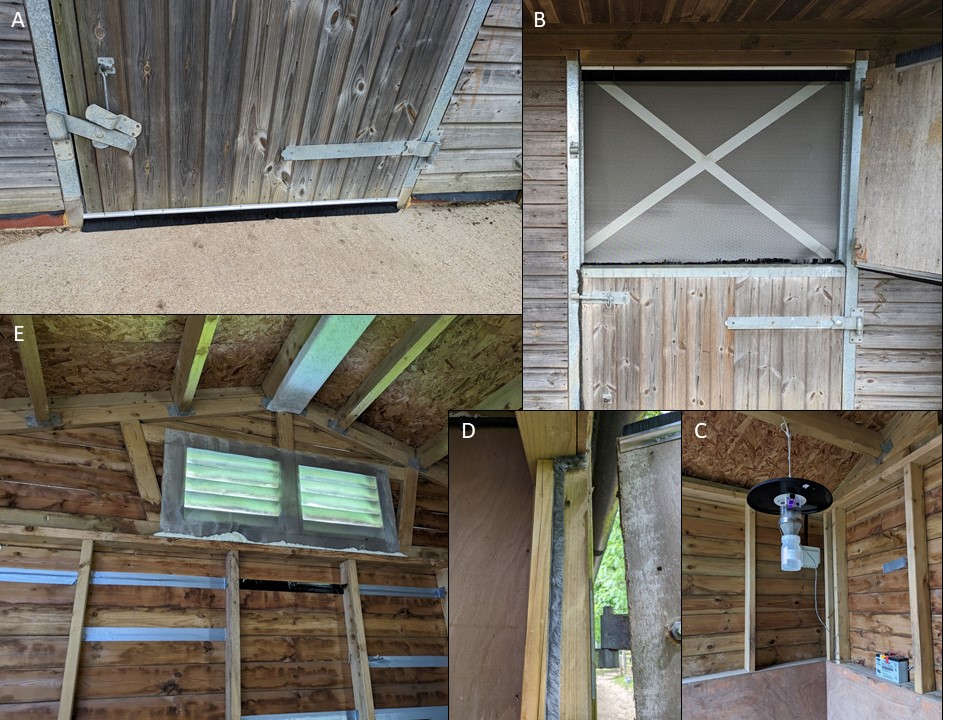

Supplement: Supplementary file 1 — Supplementary Material 1. Figure S1. Materials used to vector-proof the stables and CDC light trap within a stable. A: Five-centimetre garage door brushes, B: fine stainless steel mesh, C: CDC light trap within stable, D: felt door seal andduct tape, E: expanding foam and fine stainless steel mesh [file 13071_2025_6736_MOESM1_ESM.jpg]
