# Supplementary material for: The efficacy of vector-proof accommodation for the protection of livestock against Culicoides biting midges
Source: Parasit Vectors. 2025 Mar 14;18:108. doi: 10.1186/s13071-025-06736-9 (PMC11909808; doi:10.1186/s13071-025-06736-9)
Supplement: Supplementary file 2 — Supplementary Material 2. Table S1. Estimated coefficients in the negative binomial GLMs for numbers of Culicoides caught each trap night. [file 13071_2025_6736_MOESM2_ESM.docx]

**Table S1.** Estimated coefficients in the negative binomial GLMs for numbers of *Culicoides* caught each trap night

| Parameter | Total *Culicoides* | | | Subgenus *Avaritia* females | | | *Culicoides obsoletus*/*scoticus* females | | |
| --- | --- | --- | --- | --- | --- | --- | --- | --- | --- |
|  | Estimate | 95% confidence limits | | Estimate | 95% confidence limits | | Estimate | 95% confidence limits | |
|  |  | Lower | Upper |  | Lower | Upper |  | Lower | Upper |
| Intercept | -2.09 | -4.00 | -0.12 | -2.49 | -4.55 | -0.39 | -2.43 | -4.50 | -0.30 |
| Location |  |  |  |  |  |  |  |  |  |
| Outside (back) | 0 (Baseline) | - | - | 0 (Baseline) | - | - | 0 (Baseline) | - | - |
| Outside (front) | 1.23 | 0.21 | 2.25 | 1.47 | 0.37 | 2.57 | 1.45 | 0.34 | 2.56 |
| Stable 1 | -0.60 | -1.62 | 0.41 | -0.65 | -1.76 | 0.45 | -0.66 | -1.78 | 0.45 |
| Stable 2 | 0.02 | -1.01 | 1.04 | 0.02 | -1.10 | 1.13 | -0.07 | -1.20 | 1.06 |
| Stable 3 | -2.02 | -3.10 | -0.94 | -2.23 | -3.44 | -1.04 | -2.23 | -3.44 | -1.03 |
| Stable 4 | -2.34 | -3.45 | -1.25 | -2.53 | -3.77 | -1.32 | -2.67 | -3.94 | -1.43 |
| Temperature (°C) | 0.30 | 0.17 | 0.42 | 0.31 | 0.17 | 0.44 | 0.30 | 0.17 | 0.44 |
